# Use of Multi-Modal Data and Machine Learning to Improve Cardiovascular Disease Care

**DOI:** 10.3389/fcvm.2022.840262

**Published:** 2022-04-27

**Authors:** Saeed Amal, Lida Safarnejad, Jesutofunmi A. Omiye, Ilies Ghanzouri, John Hanson Cabot, Elsie Gyang Ross

**Affiliations:** ^1^Division of Vascular Surgery, Department of Surgery, Stanford University School of Medicine, Stanford, CA, United States; ^2^Department of Medicine, Center for Biomedical Informatics Research, Stanford University School of Medicine, Stanford, CA, United States

**Keywords:** machine learning, big data, Artificial Intelligence, cardiovascular risk factors, learning health care system, cardiovascular risk prediction

## Abstract

Today's digital health revolution aims to improve the efficiency of healthcare delivery and make care more personalized and timely. Sources of data for digital health tools include multiple modalities such as electronic medical records (EMR), radiology images, and genetic repositories, to name a few. While historically, these data were utilized in silos, new machine learning (ML) and deep learning (DL) technologies enable the integration of these data sources to produce multi-modal insights. Data fusion, which integrates data from multiple modalities using ML and DL techniques, has been of growing interest in its application to medicine. In this paper, we review the state-of-the-art research that focuses on how the latest techniques in data fusion are providing scientific and clinical insights specific to the field of cardiovascular medicine. With these new data fusion capabilities, clinicians and researchers alike will advance the diagnosis and treatment of cardiovascular diseases (CVD) to deliver more timely, accurate, and precise patient care.

## Introduction

Cardiovascular disease (CVD) is a well-known leading cause of death worldwide, accounting for almost a third of all deaths globally (1). In the United States, CVD is widely prevalent, with 1 in 3 adults documented as having some form of CVD (2), and cases have doubled to an estimated 523 million worldwide (3). It is projected that almost half of the US population will have at least one type of CVD by 2035 (4).

CVD is a major contributor to disability and is a leading cause of primary hospital admissions in the US (5), with heart failure ranking as the number 1 cause of Medicare readmissions (6). CVD is also a significant contributor to rising healthcare costs, which have continued on an upward trajectory over the past decade (3). In the US alone, the estimated financial burden of CVD is over $400 billion, which is poised to further increase due to the aging population and the increased prevalence of obesity (2). The direct medical cost of CVD is projected to grow to $749 billion in 2035, with total costs, direct and indirect, potentially crossing the $1 trillion mark for the first time ever (4).

While CVD will continue to play a crucial role in our society for the foreseeable future, recent research demonstrates that there can be considerable gains from effective CVD management. In a life table analysis, Anderson et al. show that effective management of CVD risk factors can translate into a 7-year increase in life expectancy for the US population (7). Such data demonstrate the need for tools to increase our ability to prevent and manage CVD both at the population and individual levels. The explosion in healthcare data due to the adoption of electronic medical records (EMR) and other data sources and advances in computational algorithms enable the development of technologies that automate and enhance aspects of healthcare delivery. These technologies in aggregation could improve lives and decrease dollars spent on healthcare to the tune of $600 per person due to increased health care efficiency (8).

With machine learning (ML) and Artificial Intelligence (AI), the ultimate goal is to train models using collected data to make predictions about the future, in some ways mimicking human intelligence. Traditional machine learning algorithms have focused on one data modality (e.g., imaging OR clinical text). However, this does not quite mimic human intelligence, as humans perceive environments by analyzing and integrating information from various data forms, such as image and sound. Thus, to build more robust models than those constructed based on a single modality, researchers have strived to develop algorithms that can integrate different modalities of data such as image, text, and speech. The main idea in multimodal machine learning is that different modalities provide complementary information in describing a phenomenon (e.g., emotions, objects in an image, or a disease).

Multimodal data refers to data that spans different types and contexts (e.g., imaging, text, or genetics). Methods used to fuse multimodal data fundamentally aim to integrate the data with values of different scales and distributions into a global feature space (i.e., database) in which data can be represented in a more uniform manner (9). This uniformity can then be leveraged for tasks such as prediction and classification. For example, data from large biobanks such as the UK biobank, the Million Veterans Program, and the National Institutes of Health *All of Us* initiative contain patient-specific genomic data, imaging studies, and phenotypic data from EMR and questionnaires (10–12). Each of these data types can be fused to predict cardiovascular disease prognosis, improve identification of unique subgroups, and predict response to treatment. The hope is that more accurate models can be built with multiple types of data than if only one type of data were utilized.

In other words, data fusion aims to overcome problems that arise by using only one type of data. For instance, while medical imaging provides exquisite anatomical detail, it does not contain other important information such as demographics or clinical diagnoses that can enrich clinical prediction or phenotyping tasks. Other data, such as unstructured medical records, contain rich phenotypic data but also suffer from issues of missing data and encoding medical practice rather than true biology. Such data can be combined with genetics and/or physical activity data to supplement missing data from imaging and/or unstructured medical records. However, with the exciting promise of data fusion comes interesting and important technical challenges; chief among them is transforming different data types into a format that enables efficient processing by machine learning algorithms. Though examples of multi-modal data and machine learning models in the cardiovascular space are limited, nevertheless, in this review, we highlight specific use cases focused on diagnostics, prediction, and clinical decision making (Table 1). We discuss technical considerations for data fusion modeling and conclude with recommendations for future directions.

**Table 1:** Summary of the research in cardiovascular disease care using multimodal learning.

**Model objective**	**Data modalities used**	**Learning algorithms**	**Evaluation metrics and performance**	**Citation**
Opportunistic risk assessment for ischemic heart disease	- Radiomics from abdominopelvic Computed Tomography - Electronic Medical Records data	XGBoost, an optimized gradient-boosting machine learning system	- AUROC: 0.86 - AUCPR 0.70	Chaves et al.
Improve IHD Prediction	- Electronic health records - Genetics (multiple risk loci)	Logistic regression, Random forest, gradient boosting trees, CNN, and LSTM	- AUROC: 0.790 - AUPRC 0.285	Zhao et al.
Acute coronary artery disease detection	- Electrocardiograms - Phonocardiograms - Echocardiography - Holter monitor data - Clinical lab values	Support vector machine with linear and RBF kernels	- Average accuracy: 96.67% - Sensitivity: 96.67% - Specificity: 96.67% - F1 score: 96.64%	Zhang et al.
Comprehensive noninvasive diagnostics of coronary artery disease	- Computed Tomography coronary angiography - Computed Tomography-derived fractional flow reserve - Whole-heart dynamic 3D cardiac Magnetic resonance imaging perfusion - 3D cardiac Magnetic resonance imaging late gadolinium enhancement	Fully connected neural networks	- Radiologist assessments of fused image quality: rated as good to excellent - Accuracy: highest accuracy found in revealing scars or stenoses (75%)	Von Spiczak et al.
Identify cardiovascular disease subgroups	- Genetic (SNPs) - Imaging - Demographic - Clinical - Lifestyle	Generalized low rank modeling and K-means clustering	−4 unique coronary artery disease subgroups with distinct clinical trajectories	Flores et al.
Automated cardiovascular disease detection and care recommendations	- mobile and medical sensors (respiration rate, oxygen saturation, blood pressure temperature and electrocardiograms data) - EMR (lab tests, medical history, and general medical observations)	Ensemble deep learning	- Precision: 84.5% - Recall: 84.5% - Accuracy: 82.5% - F1-measure: 83.5% - RMSE: 0.32 - MAE: 0.25	Ali et al.

*EMR, electronic medical record; RBF, radial basis function SNP, single nucleotide polymorphism; AUROC, area under the receiver operating characteristic; AUCPR, area under the precision-recall curve; IHD, ischemic heart disease; RMSE, Root Mean Square Error; MAE, Mean absolute error*.

## Multimodal Data Fusion Across Different Use Cases

### Improved Cardiovascular Disease Risk Assessment

When it comes to cardiovascular population health, the American Heart Association Pooled Cohort Equations and the Framingham coronary heart disease risk score are commonly cited tools for assessing an individual's 5–10 year risk of developing clinically significant cardiovascular disease (13, 14). Utilizing demographic and clinical data related to cholesterol and common comorbidities, these risk scores have stood the test of time as reasonable estimates of the risk of incident disease and are recommended in multiple clinical practice guidelines and policy recommendations. However, the performance of these scores, measured by the area under the receiver operating characteristic curve (AUC), has been modest when testing them in more diverse patient populations. Thus, Chaves et al. developed a framework to use deep learning and machine learning models that enable opportunistic risk assessment for ischemic heart disease (IHD) using automatically measured imaging features from abdominopelvic CT examinations in combination with information from the patient's EMR (15).

**Figure 1:**
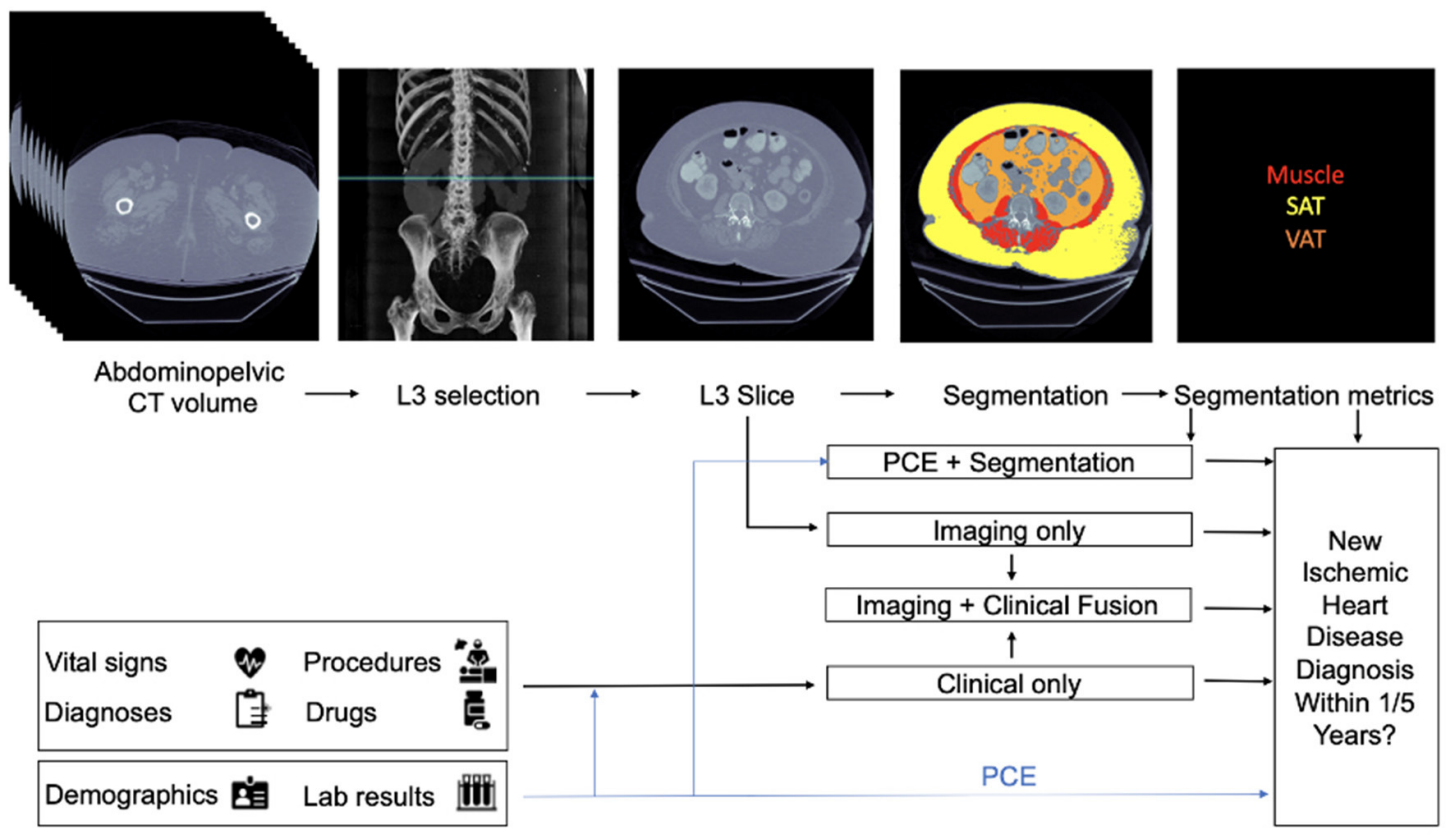
Architecture of multi-modal data fusion combining Imaging and clinical data. Figure taken from Chaves et al. (15). In their described framework, readily available CT images are combined with clinical data (e.g. vital signs, diagnoses) to predict the likelihood of ischemic heart disease at 1 and 5 years.

At a single health care institution, abdominopelvic CTs were used to extract and measure body composition (BC) biomarkers, such as hepatic steatosis, low muscle mass, and an increased ratio of visceral to subcutaneous adipose tissue. These data were combined with EMR data to develop risk models for 1- and 5-year incident IHD. Researchers collected a dataset of 8,197 CT images from individuals with at least 1 year of follow-up, and 1,762 images were obtained from 1,686 individuals who had at least 5-years of follow-up. The average length of follow-up was 3.6 years. For each individual, data available in the EMR in the year before the scan acquisition was obtained. Authors then developed four types of models (Figure 1): A Segmentation Only model, based on segmentation data from CTs, an Imaging Only model, constructed from automated features extracted from CTs, a Clinical Only model based on EMR data, and Fusion models, where all three data types (CT segmentation, automated CT extracted features, and clinical EMR data) were combined to predict IHD risk.

In the Segmentation Only model, the authors used a Convolutional Neural Network (CNN) (16), known as a 2D U-Net model, to segment body composition biomarkers, which consisted of identification of muscles, visceral adipose tissue (VAT), and subcutaneous adipose tissue (SAT). A logistic regression model was then constructed based on the extracted measurements to predict IHD outcomes at 1 and 5 years. The Imaging Only Model was constructed using the EfficientNet-B6 network (17), a CNN-based model, to predict IHD using a single slice from a CT image. Their third model (the Clinical Only model) was developed to model clinical and demographic features from the EMR within 1 year of image acquisition and included features such as demographic data, vital signs (blood pressure, heart rate, respiratory rate, oxygen saturation, temperature), body mass index, and relevant laboratory results (total, low-density lipoprotein, high-density lipoprotein cholesterol, triglycerides, fasting glucose and hemoglobin A1c). The Clinical Only model predictions were performed using an XGBoost algorithm (18). Finally, to evaluate the performance of adding imaging to different data types, three fusion models were constructed. The first fusion model was constructed based on Pooled Cohort Equations (PCE), average muscle radiodensity, and the VAT/SAT ratio from the Segmentation Only model. In the second model, features from the Imaging Only model and Clinical ones were fused. Finally, the third fusion model combined the three modalities of Imaging, Clinical, and Segmentation data.

Model performance was assessed using AUC and area under the precision-recall curve (AUCPR) metrics. Examining traditional risk factors, the PCE outperformed the FRS in 1-year IHD estimates (*P* = 0.04), but not in 5-year estimates, with AUC/AUCPRs of 0.75/0.12 and 0.71/0.09 at 1-year and 0.73/0.41 and 0.71/0.40 at 5-year, respectively. Their Segmentation Only model achieved a 1-year AUC/AUCPR of 0.70/0.08 and 5-year results of 0.73/0.43. The Imaging Only model's 1-year AUC/AUCPR was 0.74/0.10 and 0.81/0.64 for 5-year estimates, outperforming both the Segmentation Only and PCE/FRS models. Their Clinical Only model achieved similar performance to the PCE at 1-year but showed improved performance for the 5-year prediction (1-year AUC/AUCPR of 0.76/0.12, and 5-year results of 0.84/0.64). Evaluating their fusion models, the best performing model was ultimately the Imaging + Clinical 5-year model, which achieved an AUC of 0.86 and AUCPR of 0.70. Adding segmentation data to this model did not improve performance. Based on their results, the authors concluded that fusion models can be used to automate the detection of IHD risk in patients who present for care, and obtain abdominopelvic contrast-enhanced CTs for any reason.

Another example of data fusion efforts that provide a performance improvement over traditional risk factor modeling was described by Zhao et al. (19). In their efforts, researchers modeled data from the EMR combined with genetic data to predict 10-year risk of IHD. To do this, the authors evaluated EMR data within a 7-year window and built classifiers to identify risk of IHD in the following 10-year period. Feature selection was performed using Chi-square analysis (20) of the EMR data, resulting in 40 EMR-related variables. Single nucleotide polymorphisms (SNPs) were derived from 2 large meta-analysis studies, of which 204 SNPs were available in the authors' dataset. The authors compared the performance of several models including an ML model using traditional risk factors in the American Heart Association PCEs, ML models using aggregated EMR data and DL models using longitudinal EMR data. Lastly, they performed data fusion to combine SNP data in a two-stage approach. The authors first trained separate models to classify the risk of IHD—one model using only EMR data and one using only genotyped data. The predictions of these two models were then fed into an ML model for a final prediction. The final analysis included 109,490 patients in the clinical data only model (of which 9,824 were cases) and 10,162 patients included in the genotyped cohort (of which there were 2,452 cases). In general, ML models using EMR data outperformed models using a small number of traditional risk factors (AUC 0.76–0.79 vs. 0.73–0.75, respectively). In the smaller cohort with genetic data, an ML model using only PCE risk factors produced an AUC of 0.698 and AUCPR of 0.396, while an ML model utilizing longitudinal EMR data produced an AUC/AUCPR of 0.71/0.427. The addition of genetic data in their late fusion approach had a significant effect on model metrics, improving AUC/AUCPR by 2.1 and 9.1% respectively (*P* < 0.05). Zhao et al. highlight that longitudinal data better captures variability in physiological and laboratory data and are more informative in determining the risk of IHD. Furthermore, they point out the importance of including genetic variants in risk estimates for diseases with a large heritable component.

### Improved Acute Cardiovascular Disease Detection

Zhang et al. proposed an approach to detecting CAD in a more acute setting (21). Specifically, Zhang et al. were interested in testing their hypothesis that a fusion model would be able to detect the difference between those presenting with acute chest pain for cardiac and non-cardiac reasons. To do this, they combined data from electrocardiograms (ECG), phonocardiograms (22), echocardiography (ECHO), Holter monitors, and biological markers in 62 patients presenting with chest pain who ultimately underwent coronary angiography at a University Hospital. Of these patients, 32 had true CAD (including 22 with three-vessel disease). For this use case, they defined a multimodal feature set that included electrocardiogram (ECG), phonocardiogram (PCG), the results of 24-h Holter monitoring, echocardiography (ECHO), and biomarker levels (BIO). Using data from ECGs and phonocardiograms required pre-processing and included automated and manual techniques to remove noisiness from the data (i.e., denoising techniques). Time, domain, and Holter monitor features were extracted for each of these modalities. Biological features included lab values such as glucose and cholesterol levels, sodium and creatinine as well as blood cell counts. Features from ECHOs included left ventricular ejection fraction and any regional wall motion abnormalities. After deriving a total of 304 features, the authors applied a hybrid feature selection model to identify the topmost informational features in each data domain.

Once the feature selection process was completed, investigators combined data into one large feature matrix. They then evaluated the optimal number of modalities to use in their final models. Using a support vector machine algorithm with nested cross-validation, the results showed that in terms of multimodal feature models, PCG and Holter; PCG, Holter and ECG; PCG, Holter, ECG, and biomarker levels; ECG, PCG, Holter, ECHO, and biomarker levels, were the optimal bimodal, three-modal, four-modal, and five-modal models, with accuracies of 90.38, 91.92, 95.25, and 96.67%, respectively. Among them, the five-modal model, constructed by combining features from ECG, PCG, Holter, ECHO and biomarker levels, achieved the best classification results with an average accuracy, sensitivity, specificity, and F1-measure of 96.67, 96.67, 96.67, and 96.64%, respectively. Thus, the authors concluded that multimodal feature fusion and hybrid feature selection could obtain more effective information for acute CAD detection and provide a reference for physicians to improve the diagnosis of CAD patients prior to an angiogram. Whether this approach is ultimately more cost-effective than immediate coronary angiography in cases where patient chest pain etiology is ambiguous would depend on the practice setting but is promising overall.

### Improved Cardiovascular Disease Severity Assessment

**Figure 2:**
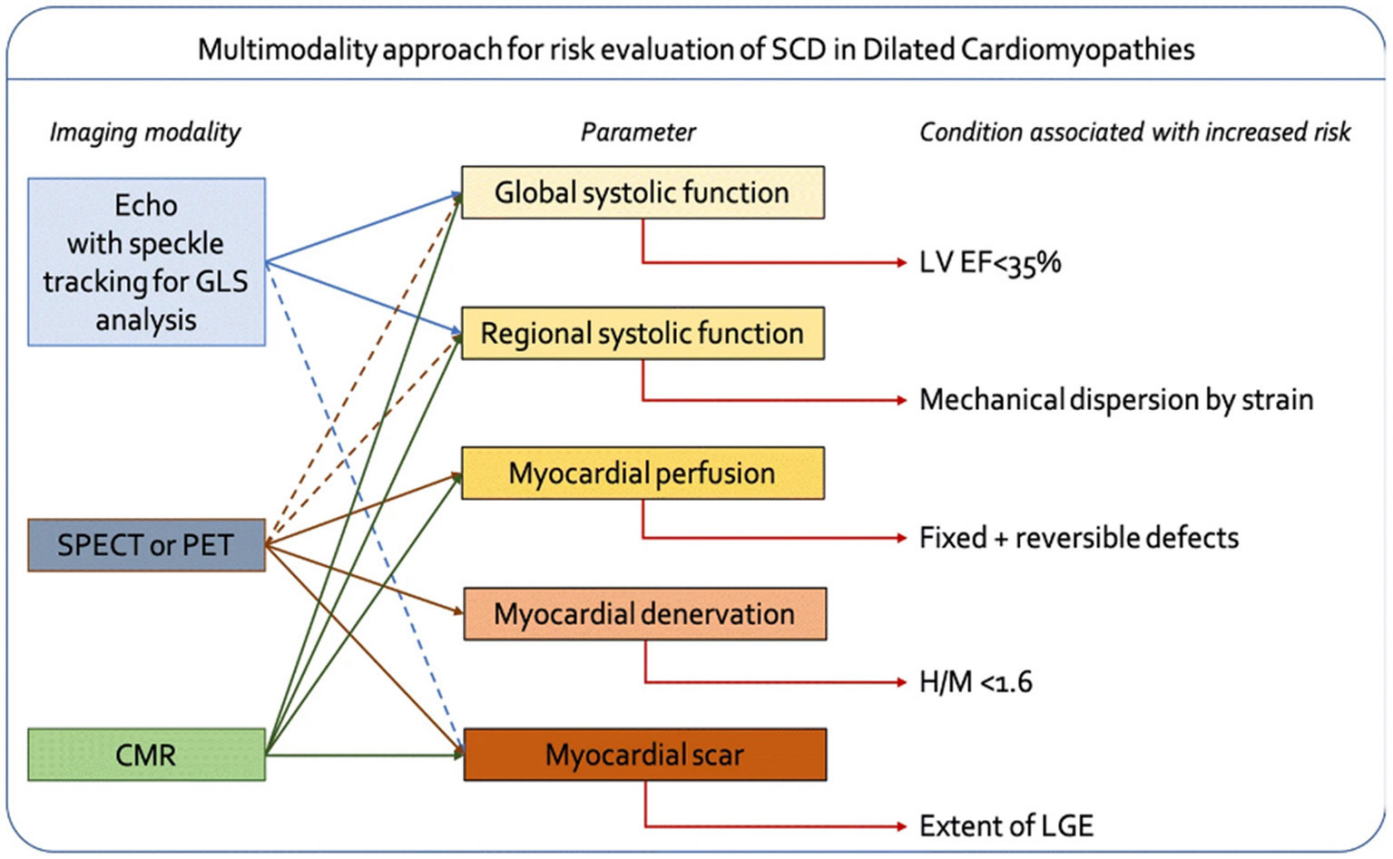
Framework for combining multiple imaging modalities to improve accuracy of predicting sudden cardiac death (SCD) in patients with dilated cardiomyopathies [from Bandera et al. (24)].

In the past 60 years, we have seen an explosion in cardiovascular imaging modalities translated to direct clinical practice (23). From 3-dimensional ECHO technology to nuclear medicine perfusion scans, clinicians have been able to derive better insights into cardiac function and structure that enables more precise clinical decision making. While each modality has its specific use case, fusing imaging modalities can increase understanding of how cardiac perfusion, structure, and function affect patient outcomes and theoretically enable better medical and surgical treatments. For example, Bandera et al. provide an overview of how multiple imaging modalities can be combined to improve the prediction of sudden cardiac death (SCD) in individuals with dilated cardiomyopathies (Figure 2) (24). While SCD accounts for over 200,000 deaths a year in the U.S. in those with cardiomyopathies, it can be difficult to predict who is at highest risk. Furthermore, pharmacological agents have been proven to reduce the risk of SCD; thus, targeting appropriate patient populations can have a significant impact on disease outcomes. As Bandera et al. point out in their review, ECHO is a gold standard for evaluating left ventricular function, and new technologies such as speckle tracking ECHO (STE) enable the opportunity to assess regional myocardial function abnormalities that might be more predictive of abnormalities that cause SCD, such as arrhythmias. On the other hand, cardiac magnetic resonance imaging (CMR) enables better characterization of important tissue characteristics such as scar formation, which is also associated with risk of SCD. Thus, combining the advantages of multiple modalities into a single model for SCD prediction may be more powerful than using each modality alone.

Another way that multimodal imaging can improve cardiovascular care is by reducing the cost and invasiveness of diagnostic studies. Healthcare services for IHD are estimated to cost >$200 billion annually in the U.S. Part of the costliness in IHD care involves invasive treatments such as coronary angiography (CA). While most non-invasive tests range from $110 to the extreme of PET at $1,500, coronary angiography generally costs an estimated $1,360–$2,810 in most U.S. health systems, depending on the place it is performed (25). As most coronary angiograms are usually accompanied by an intervention, the costs can rise to as high as $11,685. Coronary angiography, while a gold standard way to evaluate and treat CAD can also lead to higher costs, and given its invasiveness, more complications than other modalities. Thus, researchers have been working to identify ways to obtain the same diagnostic information using less-expensive, safer, non-invasive methods.

Von Spiczak et al. proposed a new framework for comprehensive noninvasive diagnostics of CAD to detect treatable lesions by using three-dimensional (3D) image fusion, merging data from CT and MRI images (26). To test their fusion framework, they performed a study on seventeen patients that underwent cardiac CT and cardiac MRI. Patients were on average aged 54 years (±10 years). All but one study participant was male. Von Spiczak et al. introduced a method of comprehensive noninvasive diagnostics for CAD that aimed to visualize multiple pathologic aspects of the disease by using multimodal multiparametric three-dimensional image fusion and advanced three-dimensional rendering techniques on different imaging modalities. By using state-of-the-art image post-processing techniques and projecting post-processed images onto a single diagram, their methodology combined CT coronary angiography, CT-derived fractional flow reserve (CT-FFR), whole-heart dynamic 3D cardiac MRI perfusion, and 3D cardiac MRI late gadolinium enhancement. When evaluating and comparing the detection capabilities across the modalities and the images outputted from the fusion model, the image quality was rated as good to excellent by two radiologists. In performing a qualitative assessment of the advantages of fusion imaging vs. individual modalities, the authors pointed to a few important examples. In one patient example, fusion imaging allowed easier correlation between visualized perfusion deficits on the MRI perfusion study and more precise localization of the etiology of this deficit, which arose from the first diagonal branch and a stented side branch. In another patient, fusion imaging allowed mapping of differences in severity of LAD stenosis with varying areas of tissue viability. Even so, Von Spiczak et al. acknowledge the small study size and complexity in acquiring and fusing imaging modalities. However, improvements in the fusion architecture that allow for more streamlined image processing and more extensive studies may enable better clinical utility and ultimately produce results that can decrease image acquisition cost and improve providers' decision making.

### Improved Cardiovascular Disease Phenotyping

Cardiovascular population health is another area in which data fusion can lead to greater insights. Clinicians intuitively know, for instance, that patients vary in socioeconomic, demographic, and clinical severity, which can require different approaches to improve disease management and outcomes. For example, some patients may require a greater focus on social determinants of health to improve outcomes in addition to adequate medical management. With this in mind, Flores et al. aimed to evaluate whether multimodal data could help provide insights into different cardiovascular phenotypes that might lend themselves to different clinical approaches (27). Previous work in the domain of cardiovascular phenotyping was described by Shah et al., who demonstrated that unsupervised learning techniques such as hierarchical clustering can be used to identify clinically meaningful subgroups of patients with CHF (28). While this helped establish unsupervised learning as a useful way to identify clinical subgroups that may benefit from different therapies, Shah et al. were limited by the data they could use. With traditional clustering models, data are typically required to be in the same format (numerical, ordinal, or categorical). Instead, Flores et al. aimed to combine genetic, imaging, demographic, clinical, and lifestyle data to identify cardiovascular disease subgroups using unsupervised methods.

**Figure 3:**
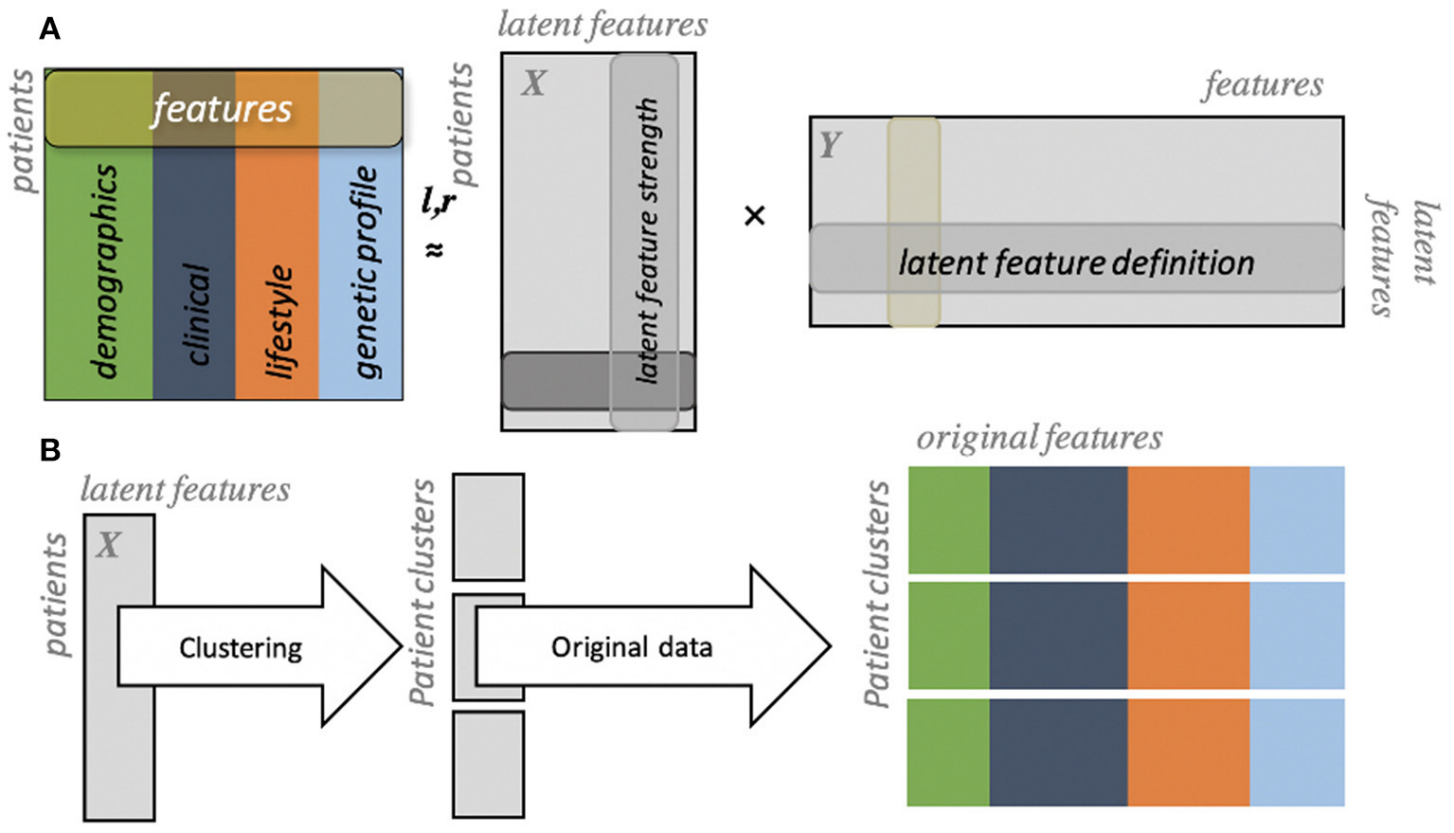
Generalized low rank modeling. **(A)** Multiple features are summarized into two low rank matrices (X and Y). **(B)** Individuals can then be clustered using latent features, after which original features can be re-identified to summarize clinical features of each group [from Flores et al. (27)].

In their efforts, Flores et al. utilized clinical trial data that consisted of over 150 variables that spanned from categorical to numerical values. Data were first summarized using a technique known as generalized low-rank modeling (GLRM) (29) which allows for the combination of multiple data types into latent features that are easier to use for unsupervised learning algorithms such as clustering (Figure 3). By applying this methodology to a subgroup of clinical trial participants with CAD, the authors identified four clinically distinct clusters of patients. One cluster included young individuals that were mostly diabetics, had low socioeconomic status and education attainment. This group was at highest risk of future major adverse cardiovascular events. Another cluster of patients included those who had a high prevalence of peripheral artery disease, were older, more likely to be previous smokers, and had the highest risk of future mortality. Lastly, the authors found two clusters that initially appeared similar—middle-aged individuals with relatively high socioeconomic status and generally better health habits than the previous two clusters. However, one cluster had a slightly higher prevalence of genetic risk markers for cardiovascular disease and higher rates of major adverse cardiovascular events than their counterparts. In addition to the insights derived from this analysis, Flores et al.'s work provides a machine learning framework by which insights from population health can be automatically derived and potentially acted upon at scale.

### Automated Cardiovascular Care Recommendations

**Figure 4:**
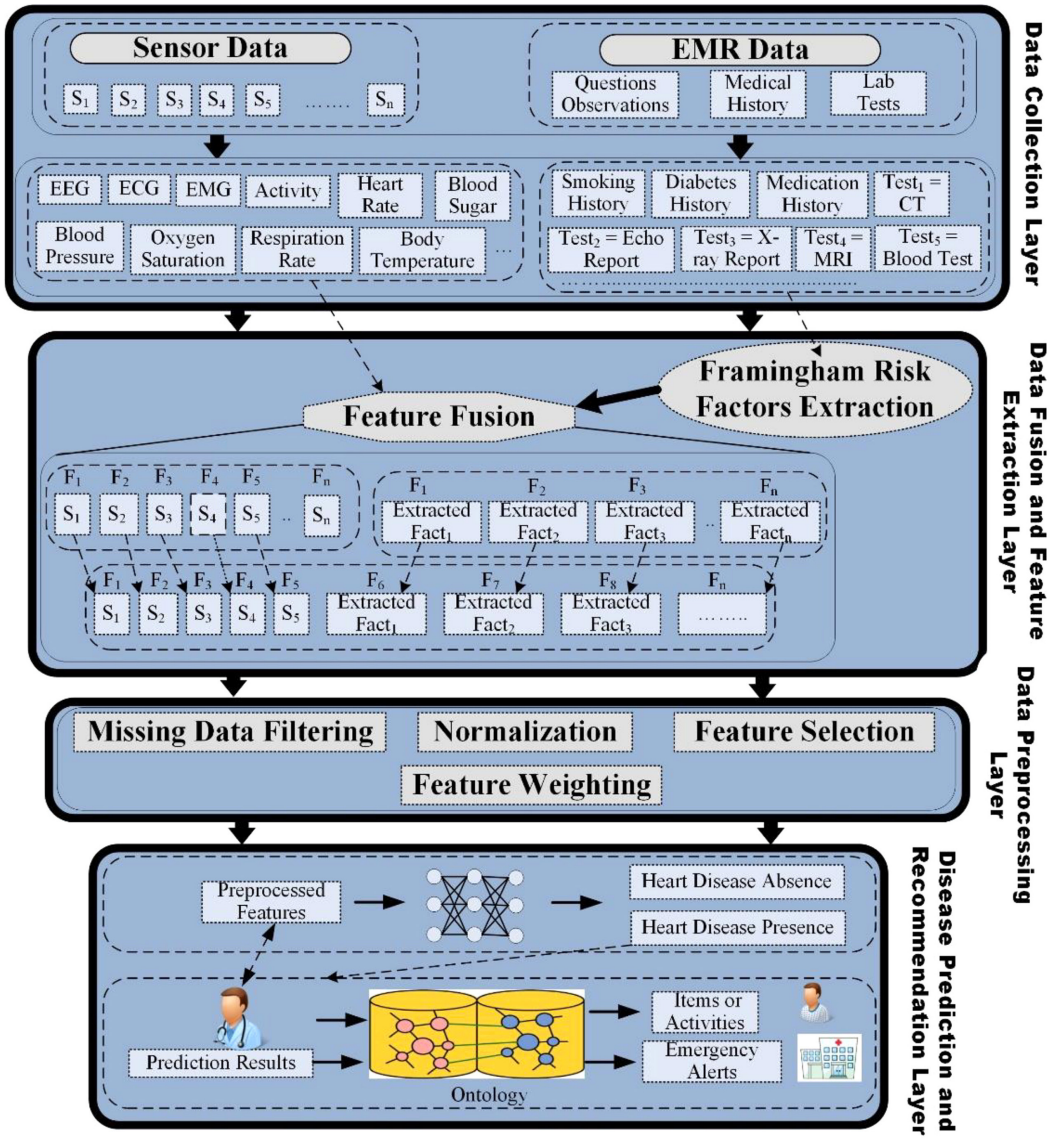
Information framework for heart disease prediction and recommendations. Figure taken from Ali et al. (31).

In addition to improved disease detection and prognosis, exciting application areas for ML and AI include contributions to a learning healthcare system whereby data from multiple sources are analyzed and used to guide treatment and lead to iterative improvements in healthcare delivery (30). Ali et al. propose a multimodal fusion model that can be used not just for detecting disease, but for making automated recommendations about cardiovascular care (31). In their framework (Figure 4), they describe a process in which multiple layers of data processing, aggregation and prediction modeling can be utilized. Specifically, the first layer involves data collection where data is extracted from multiple sources, including mobile and medical sensors that collect physiological parameters such as respiration rate, oxygen saturation, blood pressure, temperature and ECG data. Another source of data can be electronic medical records that include lab tests, medical history, and general medical observations. The second layer is the data fusion and feature extraction layer, which first involves the extraction of clinically relevant factors (such as Framingham risk factors) to estimate the risk of heart disease from unstructured data. Data fusion is then performed using the combination of data from multiple data sources into a large feature matrix. The third layer in their framework includes data pre-processing that includes four tasks—(1) data filtering, removal of duplicate and inconsistent data, and handling missing data; (2) normalization of multiple types of data distributions to between 0 and 1 to make data useable by computational algorithms; (3) feature selection that aims to reduce or eliminate noisy or redundant variables; and (4) feature weighting using conditional probability to improve the predictive accuracy. After pre-processing, in the fourth layer, a deep learning algorithm is used to make predictions regarding disease presence, or other outcomes of interest. The authors further expand on disease identification paradigms and include the possibility of including data from the literature and clinical expertise to develop a rules-based system whereby patients would be recommended to engage in certain physical activity and/or dietary plans based on their age and gender. While theoretically compelling, Ali et al. use a small subset of data to demonstrate their framework. Ultimately, richer data is needed to evaluate the utility of automated disease detection paired with rules-based treatment recommendations to enable a learning health care system.

## Data Fusion Considerations

**Figure 5:**
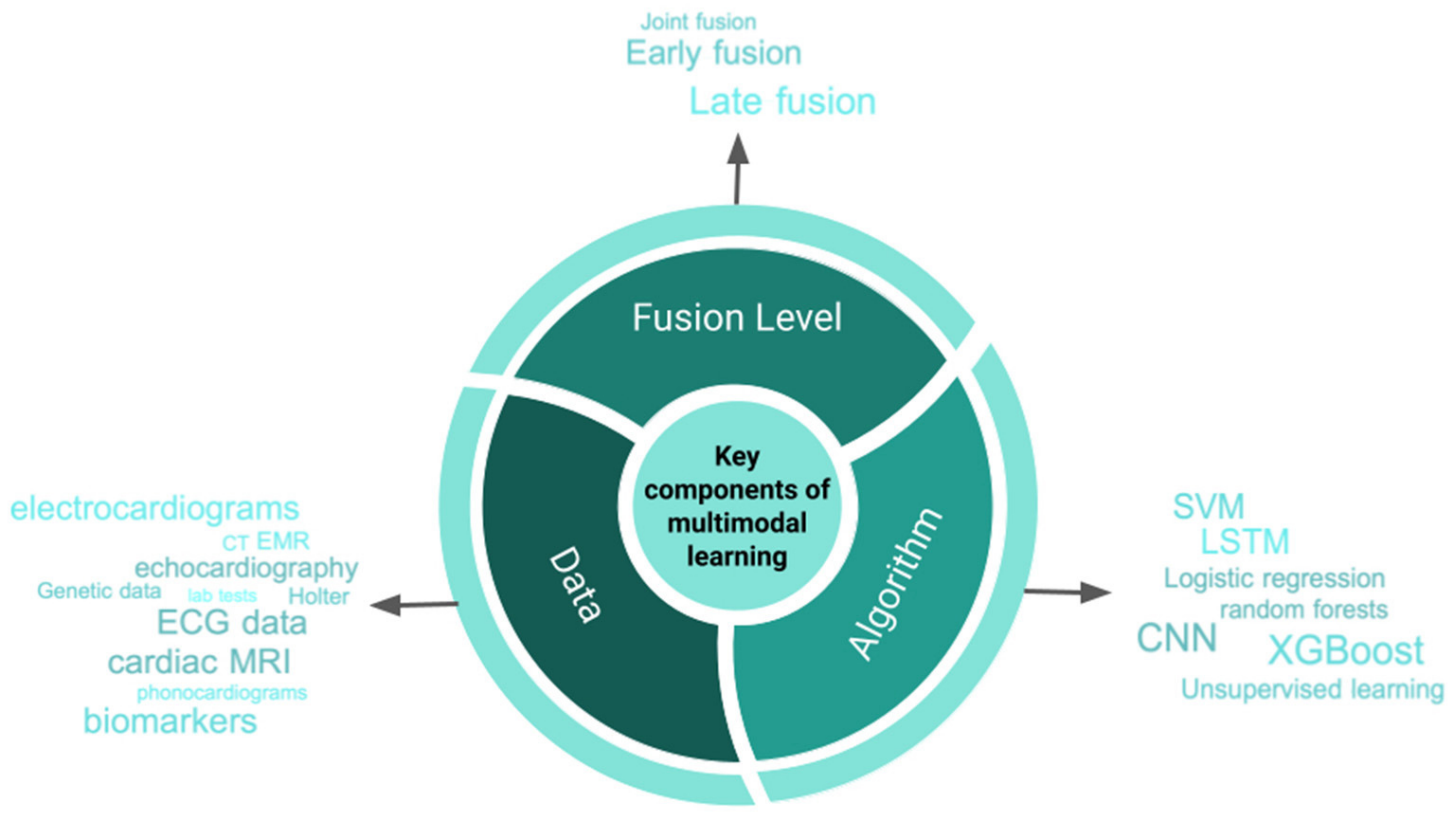
Central Illustration. Important components of developing machine learning-based models using multiple data modalities. CNN, convolutional neural networks; LSTM, long short term memory; ECG, electrocardiogram; RBF, radial basis function; SVM, support vector machine; CT, computed tomography; MRI, magnetic resonance imaging.

As detailed above, the use cases for multimodal data fusion and machine learning are varied. In Figure 5 we illustrate a distillation of the key components to developing multimodal data fusion models. In the next part of this review, we will discuss issues that should be considered when embarking on research and development that involve data fusion.

### Stages of Data Fusion

As previously mentioned, the data fusion process combines data from multiple modalities together using machine learning and/or deep learning techniques or even simpler arithmetic operations (e.g., simple concatenation). Fusion can happen at different stages of a modeling process and is mainly performed at three levels: early fusion, late fusion, or joint fusion.

Early fusion is the process of joining model features at the model's input layer mainly by combining the different types of data before applying a specific algorithm (for example, layer 2 of Ali et al.'s information framework, Figure 4). One challenge in early fusion is that it is not clear how to combine data from different modalities when the data formats are very dissimilar. As an example, consider the problem of combining tabular data (e.g., clinical biomarkers), which can be one dimensional with 3D CT imaging data. Ali et al. posit one way to address this issue using data normalization. With normalization, very different data values and distributions can be centered between 1 and 0, which allows combining data using more traditional mathematical techniques. Such an approach can also reduce data noisiness, potentially improving model predictions. Another approach is to first extract some features and measurements from each data modality and then combine this subset of features. As an example, in Chaves et al. to construct the Segmentation + Clinical fusion model, average muscle radiodensity and the VAT/SAT ratio were first extracted and then combined with clinical data (15). In contrast, for late fusion, or decision level fusion, first, for every single modality a model is trained [for example, Zhao et al.' EHR and genetic late fusion model (19)]. Next, the predictions of each model are aggregated to make a final prediction. The main downside of this approach is that none of the modalities can aid other modalities by providing any additional information since a separate model is trained for each of the modalities. Lastly, in a joint fusion approach, first, data representations are constructed for each data modality, typically using deep neural networks. All representations are then joined across modalities and fed into a prediction model. One benefit of using joint fusion compared to other fusion levels is that models can better approximate real-world interactions between data points; and thus, joint fusion can potentially lead to improved accuracy of model predictions for complex diseases or tasks (32).

### Evaluation of Data Fusion Models

Multimodal ML models are typically compared to models using fewer data modalities in order to understand what additional performance data fusion produces. Evaluation metrics, in general, are similar across ML domains and include measurements of accuracy, positive predictive value, negative predictive value, specificity, sensitivity, calibration, AUC, and AUCPR. Deciding on which evaluation metric to select mainly depends on the purpose of the study and the dataset. As an example, in classifying likelihood of myocardial infarction as a cause of chest pain, while AUC is important for understanding model discrimination abilities, health care practitioners will also need to understand model calibration—how well does a model's risk estimate match with the general risk within the population at hand? Furthermore, precision-recall metrics such as the AUCPR enable practitioners to evaluate how likely positive and negative results are to be true. Another important consideration is how well balanced the datasets used to train and test the models are. To illustrate, when studying a population of patients, it happens in many scenarios that the proportion of the patients having a particular disease is significantly smaller than those without. In this scenario, other metrics such as the F1 score, which is defined as the harmonic mean of precision and recall, provides a more fair metric than each of the two alone (precision or recall) to assess the performance of a model.

### Challenges and Opportunities

#### Challenges

Combining data from multiple sources with different intrinsic distributions and different levels of structure can be challenging. Data fusion methods aim to unify multiple data observations into a consistent and diverse representation of a phenomenon in a way that a single modality cannot provide. However, fusion itself is challenged by noisy and irrelevant data that may affect model performance, as well as missing data or scarce data, and high dimensionality (33). Additional challenges are that such a combination of data can require more sophisticated models (that can be computationally expensive to train) and more complicated data normalization techniques (which includes correction of errors and variations embedded in data from multiple sources) (34). Such model complexity can come at the cost of model “explainability.” Another issue with data fusion that Hamzah et al. point out is that it can be difficult to reconcile data that is acquired in different ways (35). For example, the quality of ECHO data is highly dependent on the expertise of the sonographer. Thus, data fidelity coming from ECHO can vary widely based on its reliance on human skill, which may affect model predictions. On the other hand, this issue points to the promise of multimodal data fusion whereby combining insights from multiple sources can supplement data that is variable.

Another challenge in working with multimodal data is that there are not good “off the shelf” techniques that will always work for any type of data combination or guarantee improved results over single modality analysis. However, algorithms such as generalized low-rank modeling (GLRM) can be considered for easier ways to combine data of different distributions and develop prediction models.

#### Opportunities and Future Directions

From a technical perspective, despite the many advances in multimodal data fusion, opportunities abound for further research. Specifically, data fusion for medical imaging is still cumbersome, as detailed by Von Spiczak et al. (26). More efficient algorithms may be needed to make fusion easier and faster to implement in order to make clinical applications a reality. Some researchers, such as Piccinelli et al., have focused on developing more efficient fusion techniques, precisely to improve clinical translation (36). Some of this improvement comes from representation learning for image analysis that enables automated image segmentation, resulting in faster fusion image rendering. Improved model prediction speeds will be key to enabling real-time predictions, which are especially important in use cases for which more urgent decisions must be made. Additional future research directions should include developing novel intuitive frameworks for investigators to understand the information gain or loss from different data modalities. While multimodal data fusion can produce better performing models, this is not always the case, thus a better framework for evaluating the utility of data modalities will help researchers focus their efforts.

From a data perspective, a focus on data quality can improve model predictions and ultimately help researchers better realize the promise of AI applied to healthcare. While there has been less focus on standards for reporting data quality to date, new standards of reporting are being operationalized (37). Focusing on improving data quality is as important as technology development for multiple reasons, the most important of which are research reproducibility and generalizability. In addition to the quality of data, the relevance of the model and effective comparison to standards of care should be considered when developing data fusion technologies, as this can significantly affect model adoption. Lastly, future research directions should focus on prospective studies comparing differences in care derived from multimodal fusion modeling compared to conventional modeling or current standards of care, as this can provide additional validation for the utility of fusion modeling.

## Conclusions

Multimodal data fusion and machine learning in cardiovascular medicine is an exciting field of research, though, there are still very few use cases to date. Using data from multiple modalities offers the promise of improved AI technology whereby the weaknesses of each type of health care data can be addressed through different data combinations. However, algorithms used to analyze multiple data modalities may be too complex, too difficult to implement, and too slow to fit into a time frame that makes them usable in a clinical work environment. Furthermore, a focus on data quality will be essential to prevent exponentially propagating errors when combining data. Future research should focus on streamlined methods for data integration, best practices for evaluating model gain from different types of data, and prospective study designs to validate clinical utility.

## Author Contributions

SA performed literature review and drafted the manuscript. LS drafted the manuscript and provided significant edits. JO drafted the manuscript and provided meaningful edits. IG and JC provided meaningful edits to the manuscript. ER conducted literature review, provided significant edits, and supervised the manuscript. All authors contributed to the article and approved the submitted version.

## Funding

ER acknowledges support from the National Institutes of Health, National Heart, Lung, and Blood Institute (K01HL148639-02) and the Doris Duke Charitable Foundation's 2021 Clinical Scientist Development Award. The funders were not involved in any aspect of this study.

## Conflict of Interest

The authors declare that the research was conducted in the absence of any commercial or financial relationships that could be construed as a potential conflict of interest.

## Publisher's Note

All claims expressed in this article are solely those of the authors and do not necessarily represent those of their affiliated organizations, or those of the publisher, the editors and the reviewers. Any product that may be evaluated in this article, or claim that may be made by its manufacturer, is not guaranteed or endorsed by the publisher.
